# Plasma branched-chain and aromatic amino acid concentration after ingestion of an urban or rural diet in rural Mexican women

**DOI:** 10.1186/s40608-015-0038-4

**Published:** 2015-02-22

**Authors:** Adriana M López, Lilia G Noriega, Margarita Diaz, Nimbe Torres, Armando R Tovar

**Affiliations:** Departamento de Fisiología de la Nutrición, Instituto Nacional de Ciencias Médicas y Nutrición, Salvador Zubirán, México

**Keywords:** BCAA, Tyrosine, Plasma amino acids, Mexican diet

## Abstract

**Background:**

People living in rural areas are prone to move to urban cities experiencing a dramatic change in the type of protein consumed. However, it is not know if those changes are associated with changes in the plasma amino acid concentration, especially the branched chain amino acids. Thus, the aim of the present study was to evaluate, in a rural Mexican population, the plasma amino acid profile after consumption of typical Mexican rural or urban diet.

**Results:**

We evaluated the plasma amino acid concentrations of women from a rural population at 0, 30, 60, 90, 120, 180 and 240 min after ingestion of a typical Mexican rural or urban diet. Ingestion of a Mexican urban diet induced a higher increase in leucine, isoleucine, valine, phenylalanine, tyrosine and proline than ingestion of a Mexican rural diet in women from a Mexican rural population. Arginine, histidine, lysine, threonine, alanine, glycine and serine had the same area under the curve regardless of the experimental diet.

**Conclusions:**

Ingestion of a Mexican urban diet induced a higher increase in leucine, isoleucine, valine, phenylalanine, tyrosine and proline than ingestion of a Mexican rural diet in women from a Mexican rural area.

## Background

Plasma branched chain amino acids (BCAA), leucine, isoleucine and valine, are increased during obesity [1-3] and this increase is associated with a 5-fold higher risk for development of type 2 diabetes [4]. Studies with animal models and humans have demonstrated that the increase in BCAA is result of a defect in BCAA oxidation due to a decrease in BCAT2 and BCKDH expression in adipose tissue [2,5]. However, there are two other possible mechanisms that can also contribute to differences in plasma amino acid concentration: 1) An increase in protein consumption; especially for BCAA and other indispensable amino acids since they are not synthesized de novo in mammalian tissues; and 2) The type of protein that is ingested; for example, consumption of an animal protein by human subjects such as lactalbumin produced a higher increase in plasma tryptophan concentration than consumption of a plant protein such as gluten or zein [6].

In Mexico, the consumption of the type of protein consumed varies according to the geographic, ethnic, cultural and socioeconomically status [7]. In the center of the country, especially in rural areas with low income, the diet is based on corn tortillas, beans, some wheat pasta, and a variety of fruits and vegetables, with little or sporadic consumption of animal products. Conversely, the urban middle-class diet includes more animal foods and refined products. Interestingly, ingestion of these two diets induces different changes in plasma amino acid concentrations throughout the day [8]. Furthermore, people living in rural areas are prone to move to urban cities looking for better opportunities and this is associated with a dramatic change in the feeding behavior that includes changes in the type of protein consumed [9]. However, it is not know if those changes are associated with changes in plasma amino acid concentrations, especially the branched chain amino acids.

Therefore, the aim of the present study was to evaluate, in a rural Mexican population, the plasma amino acid profile after consumption of typical Mexican rural or urban diet. Interestingly, the results show that ingestion of a Mexican urban diet induces a higher increase in plasma branched-chain amino acid, as well as phenylalanine, tyrosine and proline, concentration than ingestion of a Mexican rural diet in women from a rural area. These results suggests that, in the long term, changes in feeding behavior that accompanies the immigration of women from a rural to an urban area may contribute in part to the development of obesity and diabetes through modification of tyrosine and branched chain amino acid concentrations.

## Methods

### Subjects

Fifteen healthy, nonpregnant, adult women living in a rural community named Solis located in the State of Mexico volunteered for the study, which was carried out in a metabolic unit in the clinic of the community. The median (range) age of the subjects was 29 (18–49) y, body weight was 55.8 (46–82.6) kg, height was 1.57 (1.51-1.67) m, and body mass index was 22.9 (19.3-31.7) kg/m^2^. None were consuming any medication, including over-the-counter drugs, oral contraceptives or vitamins. None were in their menstrual period at enrollment or during the study. Accordingly to a 24-hours food recall, the habitual diets of these women has a ratio of vegetable to animal proteins of 2.2 and a ratio of complex to simple carbohydrates of 17.4. The mean energy intake of these women was 1413 ± 465 kcal/d. The subjects were fully informed about the purpose and design of the study and they gave written consent. The protocol was approved by the Committee of Experimental Studies in Humans from the Instituto Nacional de Ciencias Médicas y Nutrición Salvador Zubirán.

### Experimental diets and study design

The study was conducted in two periods with a crossover design. All women were asked to eat a single meal according to two different diets, a typical Mexican rural or urban diet, on two different occasions with one week in between. Diets have been previously described and where designed using a 24 hour food recall analysis of women from rural and urban areas [10]. Briefly, food composition of the typical Mexican rural diet consisted of red tomatoes (37.5 g), green chilli (12.5 g), onion (21.25 g), corn tortilla (180 g), boiled beans (112 g), and a corn-dough beverage called atole (500 ml); food composition of the typical Mexican urban diet was cantaloupe (50 g), banana (50 g), refined wheat flour as bread (100 g), pork ham (100 g), cheese (80 g), milk (300 ml), sugar (10.2 g), soluble coffee (2 g), and a cinnamon roll (64 g); the nutrient content and amino acid composition of each diet is shown in Table 1. Women were admitted at 07.00 am to the metabolic unit following a 12 h overnight fast. A catheter was placed in an antecubital vein of the arm and a baseline blood sample was taken at time 0 before consumption of the respective diet. Blood samples were taken at 30, 60, 90, 120, 180 and 240 min after ingestion of the diet.

**Table 1:** **Nutrient content and amino acid composition of the Mexican urban and rural diet**

	**Urban diet**	**Rural diet**
Energy (kcal)	1330.73	1350.14
Carbohydrates^*^	42.38	73.38
Proteins^*^	23.97	7.93
Lipids^*^	33.86	21.85
Total proteins (g)	79.76	26.77
Animal^*^	13.15	0.0
Vegetable^*^	10.83	7.93
Carbohydrates (g)	141.00	247.69
Simple^*^	39.64	22.25
Complex^*^	2.73	51.12
Lipids (g)	50.07	32.78
Animal^*^	18.50	0.0
Vegetable^*^	15.36	21.85
Dietary fiber (g)	6.95	39.22
Amino acids (g)		
Leucine &	4.79	3.68
Isoleucine &	2.74	1.70
Valine &	3.22	1.73
Arginine &	1.58	1.94
Histidine &	1.23	1.01
Phenylalanine &	2.75	1.80
Lysine &	3.33	2.09
Threonine &	2.10	1.45

^*^Percentage of total energy per diet.

& According to the Mexican food composition tables [11].

### Biochemical analysis

Plasma glucose, total cholesterol, triglycerides and HDL-cholesterol were measured enzymatically on an automated Synchron CX autoanalyzer (Beckman, CA, USA). Amino acid analysis was performed as previously described [8]. Briefly, 50 mg sulfosalicylic acid was added to 1 mL plasma to deproteinize the sample. Samples were mixed and centrifuged at 2400 X g at 4°C for 20 min and the supernatant was filtered through a filter (0.22 μm pore diameter; Millipore, Milford, MA). Samples were stored at −70°C until analyzed. Determinations were carried out in a Beckman amino acid analyzer (model 119 CL; Beckman Instruments, Palo Alto, CA) using L-Norleucine as internal standard.

### Statistical analysis

Results are presented as mean ± SEM of the 15 women that participated in the study. The area under the curve (AUC) was calculated using GraphPad Prism 5.00 (San Diego, CA). A student t-test was used to evaluate differences between AUC from urban and rural diets using the same program. Asterisk indicates a statistical difference at p < 0.05.

## Results

### Biochemical parameters

We analyzed some biochemical parameters that are modified during obesity including, glucose, total cholesterol, triglycerides and HDL-cholesterol, in our subjects (Table 2). In basal conditions, subjects showed normal values of glucose, total cholesterol and triglycerides; however, they had low HDL cholesterol according to the ATPIII. The response of glucose and total cholesterol were similar after ingestion of both diets since there were no differences in the AUC; nonetheless, after three hours of consumption of the urban diet, the glucose and total cholesterol showed a significant increase compared to the rural diet. Interestingly, consumption of rural diet presented a significant lower AUC of triglycerides, and plasma levels where significantly different particularly after 90 minutes of consumption. In our population, HDL-cholesterol concentration was low according to the ATPIII and it was not affected by the consumption of any of the diets.

**Table 2:** **Plasma glucose, cholesterol, triglycerides and HDL-cholesterol concentrations in rural women after ingestion of an urban or rural diet**

**Time (min)**	**Glucose (mg/dL)**		**Cholesterol (mg/dL)**		**Triglycerides (mg/dL)**		**HDLcholesterol (mg/dL)**	
	**Urban diet**	**Rural diet**	**Urban diet**	**Rural diet**	**Urban diet**	**Rural diet**	**Urban diet**	**Rural diet**
0	84.8 ± 3.9	82.2 ± 2.1	144 ± 10	138 ± 7.8	144 ± 31	98.2 ± 17	23.1 ± 2.5	31.1 ± 8.0
30	85.3 ± 6.3	93.1 ± 6.3	141 ± 14	147 ± 7.3	109 ± 27	112 ± 15	25.9 ± 1.9	24.0 ± 5.3
60	89.9 ± 9.4	91.7 ± 9.5	145 ± 14	140 ± 9.2	145 ± 28	123 ± 19	23.8 ± 2.4	28.3 ± 6.2
90	89.7 ± 7.8	86.3 ± 6.7	148 ± 13	141 ± 8.5	205 ± 41	122 ± 17*	24.8 ± 3.0	30.2 ± 5.7
120	90.3 ± 8.0	83.8 ± 6.1	141 ± 13	142 ± 8.7	180 ± 33	149 ± 21	27.4 ± 2.6	38.3 ± 5.2*
180	84.7 ± 16.6	80.5 ± 4.7	173 ± 15	144 ± 8.4*	361 ± 66	139 ± 22*	27.8 ± 3.1	29.3 ± 5.4
240	94.2 ± 8.8	75.4 ± 1.9*	175 ± 16	141 ± 6.9*	275 ± 53	133 ± 20*	28.3 ± 4.7	30.6 ± 2.6
AUC	21190 ± 1378	20230 ± 763	37170 ± 1928	34210 ± 1139	53910 ± 6009	31260 ± 2707*	6337 ± 408	7338 ± 735

Data are presented as mean ± SEM.

Asterisk indicates a statistical difference vs urban diet, at p < 0.05 using a t-test.

### Plasma amino acid concentration

**Figure 1:**
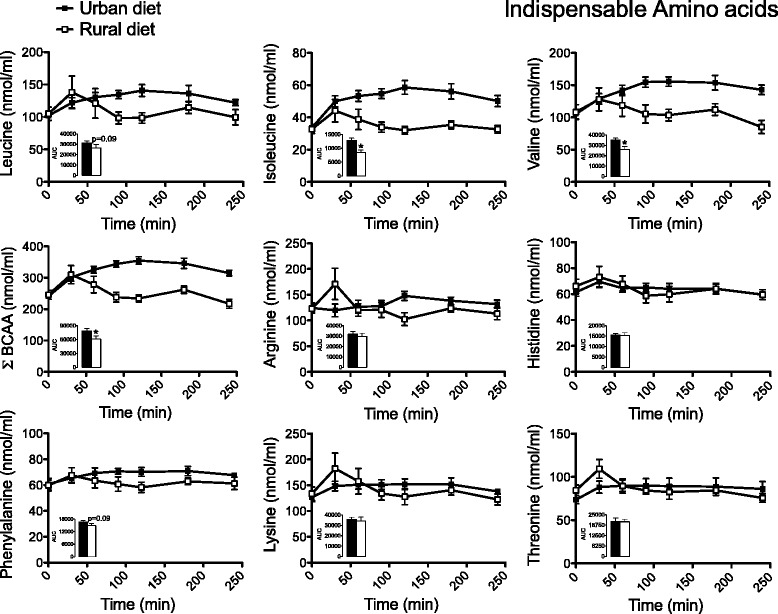
**Plasma indispensable amino acid concentrations in rural women after ingestion of an urban (black squares) or rural diet (white squares).** Blood samples were taken after 0, 30, 60, 90, 120, 180 and 240 min of ingestion of the diet. Data are presented as mean ± SEM. Asterisk indicates a statistical difference at p < 0.05 between the areas under the curve (AUC) from rural and urban diets using a t-test.

**Figure 2:**
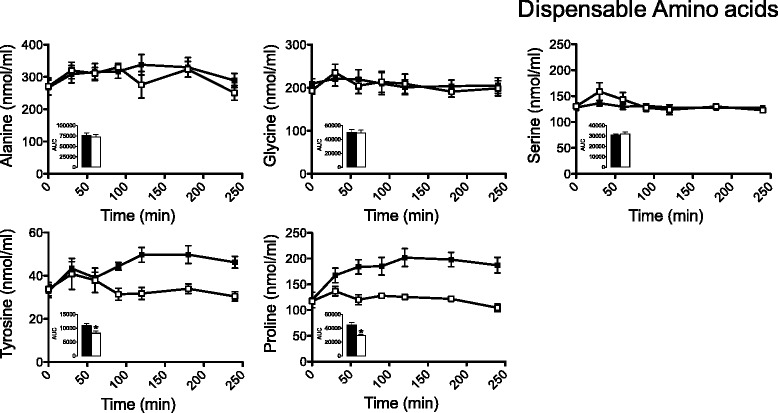
**Plasma dispensable amino acid concentrations in rural women after ingestion of an urban (black squares) or rural diet (white squares).** Blood samples were taken after 0, 30, 60, 90, 120, 180 and 240 min of ingestion of the diet. Data are presented as mean ± SEM. Asterisk indicates a statistical difference at p < 0.05 between the areas under the curve (AUC) from rural and urban diets using a t-test.

Fasting plasma amino acid concentrations were not different between testing days. For indispensable amino acids (Figure 1), the area under the curve (AUC) of leucine, isoleucine and valine was respectively 15 (p = 0.09), 33 (p < 0.05) and 25% (p < 0.05) higher when they ingested a typical Mexican urban diet than when they received the typical Mexican rural diet. In fact, the sum of plasma total BCAA concentration showed a 22% (p < 0.05) increase when women consumed the urban versus rural diet. Furthermore, we observed an increase in plasma phenylalanine and tyrosine however, only the tyrosine increase was significant (Figure 2). Interestingly, the AUC of other indispensable amino acids such as arginine, histidine, lysine and threonine showed no difference among the experimental diets. Regarding the dispensable amino acids (Figure 2), alanine, glycine and serine had the same AUC regardless of the experimental diet; and interestingly, the AUC of proline was 34% (p < 0.05) higher when they ingested a typical Mexican urban than when they received the typical Mexican rural diet.

## Discussion

Our results show that ingestion of an urban diet induces a higher increase in the plasma concentration of leucine, isoleucine, valine, phenylalanine, tyrosine and proline than ingestion of a rural diet in Mexican women from a rural area. A strength of this study is the fact that the same individuals where tested with both diets which decrease the effect of individual variation. Interestingly, the variations of BCAA and tyrosine were not directly associated with changes in plasma glucose and total cholesterol after the consumption of both diets. However, we observed that the elevation of BCAA and tyrosine with the urban diet was parallel to changes in serum triglycerides. Nonetheless, subjects fed the urban diet tended to increase plasma glucose concentration in the last hour of the study compared with subjects fed the rural diet. It is important to point out that with our study we cannot rule out that, in addition to the change in amount and type of protein, the presence of other components of the diet such as the amount of carbohydrates, fat and dietary fiber could directly impact glucose levels, insulin signaling or insulin secretion; for example, the urban diet has a higher proportion of simple carbohydrates, which have a higher insulinemic and glycemic index that can per se contribute to the development of insulin resistance. Further studies are need to assess the short- and long-term effects of the consumption of this diets on serum insulin and GLP1 levels to closely associate the changes in BCAA with insulin secretion and signaling. In addition, it is necessary to understand whether genetic variability between rural and urban population in Mexico as well as environmental conditions and physical activity can modify the biological response to the type of diet consumed.

Although the amount of protein consumed in the urban diet is greater than in the rural diet, why only these amino acids are increased requires further investigation. We can enumerate four possible mechanisms: 1) the presence of other components of the diet such as the amount and type of fiber that could alter the digestibility of the protein; 2) the fact that the first step of branched chain amino acid catabolism is extrahepatic which could delay their clearance from plasma; 3) an animal or vegetable based diet alters the gut microbiome [12], and this could modify the bacterial synthesis of branched chain amino acids; and 4) the rate at which each amino acid is incorporated in protein synthesis, i.e.: leucine is incorporated to proteins at a higher rate than isoleucine and valine.

Furthermore, there is controversy about the health implications of branched chain amino acids. In one hand, leucine activates the mTOR-S6K pathway, which inhibits the insulin receptor substrate 1(IRS1), thus, the over stimulation of this pathway by a high intake of BCAA leads to insulin resistance [3]. On the other hand, there is also evidence that high leucine intake can improve insulin sensitivity (reviewed by [13]. Our results suggest that this potential controversy depends on several factors that could contribute to such differences: 1) the source, i.e. the type of protein, vs the supplementation with BCAA or leucine, or 2) the context, i.e. a rich source of protein administered alone [14] or together with fat or complex/simple carbohydrates. In addition, beyond this controversy, it is not clear whether changes in BCAA are cause or consequence of insulin resistance.

Finally, we also observed an increase in plasma aromatic amino acid concentrations phenylalanine and especially tyrosine. An increase in serum tyrosine levels is accompanied by a concomitant increase in brain tyrosine concentration and catecholamines such as Norepinephrine (NE) [15]. As NE modulates food intake, then it is possible that the increase in tyrosine levels could modify food intake behavior. Intriguingly, we also observed an increase in proline after ingestion of the urban diet, however the biological significance of this increase is unclear. Therefore, the greater increase in both BCAA and tyrosine concentrations, could explain in part why immigrants are more susceptible to changes in body fat distribution [16] and to develop diseases related to over-nutrition such as obesity and diabetes [17,18]. In addition to immigrants, changes in feeding behavior affect rural populations. Nowadays, the food system in an increasing proportion of rural areas across low- and middle-income countries has changed drastically with the enormous penetration of super- and mega- market companies that increase the access to energy dense food [19]. In Mexico, this is reflected in the greater increase in the prevalence of overweight and obesity in rural (3.9%) compared to urban (2.5%) women in the last 6 years [20].

## Conclusions

Ingestion of a Mexican urban diet induces a higher increase in leucine, isoleucine, valine, phenylalanine, tyrosine and proline than ingestion of a Mexican rural diet in Mexican women from a rural area.
